# Primary cilia respond to fluid shear stress and mediate flow-induced calcium deposition in osteoblasts

**DOI:** 10.1096/fj.13-231894

**Published:** 2014-01

**Authors:** Robin M. Delaine-Smith, Anuphan Sittichokechaiwut, Gwendolen C. Reilly

**Affiliations:** * Kroto Research Institute, Department of Materials Science and Engineering, University of Sheffield, Sheffield, UK; and; † Periodontology Section, Department of Preventive Dentistry, Naresuan University, Phitsanulok, Thailand

**Keywords:** mechanotransduction, oscillatory fluid flow, osteogenesis, extracellular matrix

## Abstract

Bone turnover *in vivo* is regulated by mechanical forces such as shear stress originating from interstitial oscillatory fluid flow (OFF), and bone cells *in vitro* respond to mechanical loading. However, the mechanisms by which bone cells sense mechanical forces, resulting in increased mineral deposition, are not well understood. The aim of this study was to investigate the role of the primary cilium in mechanosensing by osteoblasts. MLO-A5 murine osteoblasts were cultured in monolayer and subjected to two different OFF regimens: 5 short (2 h daily) bouts of OFF followed by morphological analysis of primary cilia; or exposure to chloral hydrate to damage or remove primary cilia and 2 short bouts (2 h on consecutive days) of OFF. Primary cilia were shorter and there were fewer cilia per cell after exposure to periods of OFF compared with static controls. Damage or removal of primary cilia inhibited OFF-induced PGE_2_ release into the medium and mineral deposition, assayed by Alizarin red staining. We conclude that primary cilia are important mediators of OFF-induced mineral deposition, which has relevance for the design of bone tissue engineering strategies and may inform clinical treatments of bone disorders causes by load-deficiency.—Delaine-Smith, R. M., Sittichokechaiwut, A., Reilly, G. C. Primary cilia respond to fluid shear stress and mediate flow-induced calcium deposition in osteoblasts.

Bone turnover and homeostasis are regulated by mechanical forces, and it is predicted that bone cells *in vivo* are exposed to shear stresses originating from interstitial oscillatory fluid flow (OFF; refs. 1, 2). It has been clearly demonstrated that bone cells respond to OFF *in vitro*, and exposure of osteoblasts to OFF causes the up-regulation of a number of factors involved in bone matrix regulation, including cyclooxygenase 2 (COX-2) and osteopontin (OPN) mRNA and prostaglandin E_2_ (PGE_2_) secretion (3–5), as well as stimulating extracellular matrix (ECM) deposition (6).

Until recently, little was known about how bone cells are able to convert an external mechanical stimulus into a biochemical signal and how this relates to *in vivo* mechanisms of load-induced bone formation. A number of possible mechanisms have been identified, including mechanosensitive calcium channels (1), integrins (7), G-coupled protein receptors (8), and the cell glycocalyx [a pericellular glycosaminoglycan (GAG)-proteoglycan (PG) layer; refs. 2, 9]. However, recent work has identified the primary cilium as a promising candidate mechanosensor in bone cells (5, 10, 11).

The primary cilium is an immotile microtubule-based organelle, found one per cell, which develops from the mother centriole of the centrosome. It is anchored by the basal body and then extends like an antenna from the apical cell surface, continuous with the cell membrane. Primary cilia have been seen to exist in most mammalian tissue types and usually form several hours after growth arrest when cultured *in vitro*, taking several days to grow to full size (12), with cell spatial confinement being a major regulator of ciliogenesis (13). They are populated with receptors that participate in numerous signaling events (14) and play important roles in chemosensation (15, 16), thermosensation (17), and mechanosensation (18), and are believed to act as sensors to fluid flow. Application of a continuous 0.036 Pa flow caused primary cilia to deflect in osteoblasts (10), while the absence of a healthy primary cilium in osteoblasts and osteocytes resulted in a loss of the OFF-induced increases in OPN and COX-2 mRNA that are typically seen in the same cells when possessing a healthy primary cilium. In another study, the conditioned media of OFF-stimulated osteocytes caused a significant up-regulation of OPN and COX-2 mRNA by mesenchymal stem cells (MSCs) compared to statically conditioned medium. However, inhibition of primary cilia formation in the osteocytes prior to OFF exposure inhibited this effect (5).

While previous studies have shown that primary cilia mediate the up-regulation of specific osteogenic genes in response to OFF (5, 10), so far there have been no *in vitro* studies concerning the role of the primary cilia and load-induced mineral matrix deposition, which is the end stage of bone differentiation. The relationship between early and late responses to external mechanical stimuli is not clear, and therefore load-induced up-regulation of early osteogenic markers is not necessarily followed by a load-induced increase in matrix deposition. Chondrocyte primary cilia have been shown to be essential for cartilage matrix synthesis (sulfated GAG) in response to compressive strain as well as up-regulation of early markers associated with chondrogenesis (19).

The cilium's mechanosensory ability appears to be related to its structural properties, and it has been seen that primary cilia are able to regulate their length in response to the extracellular environment in an apparent attempt to adjust their sensitivity (20, 21). Overloading of chondrocytes resulted in a decrease in cilia length (20) and strain deprivation in tendon cells resulted in a significant cilia length increase, which was reduced on application of load in a strain-dependent manner (21). Prior to this study, it was not known whether bone cell primary cilia also adjust their length in response to load.

The deflection of the primary cilium under low fluid shear stress (FSS; 0.036 Pa; ref. 10) indicates it is able to sense low-magnitude external stimuli. We have shown previously that subjecting cells to OFF on a simple rocking platform that produces low-magnitude oscillatory FSS (peaking at 0.063 Pa) enhances extracellular mineral deposition by mature osteoblasts (MLO-A5; ref. 6). MLO-A5 osteoblasts/preosteocytes are an ideal bone cell with which to study the role of the primary cilia as a mechanotransducer in the modulation of mineralized matrix deposition, due to their rapid and robust response to short bouts of loading (9, 22). The mineral deposited by MLO-A5 cells has been well characterized using energy dispersive spectrometry and a variety of high resolution microscopy techniques and has been shown to be more like the mineral found in bone than other osteoblastic cells such as MC3T3-E1 cells (23).

There are two commonly used methods for primary cilia removal *in vitro*: application of the drug chloral hydrate (CH), which breaks down microtubules (18); or siRNA-mediated depletion of the intraflagellar transport (IFT) component polaris (10), which is required for primary cilia biogenesis and function (24). Polaris knockout prevents primary cilia from forming, while CH removes existing primary cilia and stops the formation of new primary cilia while the drug is still present. CH treatment has been shown to be effective for primary cilia removal in a number of different cell types (10, 17, 25), but can take between 24 and 96 h to completely remove all primary cilia. However, between the beginning of the application of CH and the time at which primary cilia are removed, damage is caused to cilia microtubules, which results in them shrinking and bending. This allows for investigation of the effects of a range of cilium defects, from partially damaged to completely removed, for further understanding of its function.

The aim of this work was to further investigate the role of the primary cilium as a mechanotransducer in osteoblasts. The two main objectives were first to observe whether OFF stimulation affected primary cilia morphology and second to elucidate whether primary cilia inhibition affected OFF-induced extracellular calcium deposition.

## MATERIALS AND METHODS

### Cell culture

MLO-A5 mature osteoblast cells, kindly donated by Dr. Lynda Bonewald (University of Missouri, Kansas City, MO, USA) under a Material Transfer Agreement with the University of Texas (Austin, TX, USA), were used between passages 25–28. Cells were expanded in basal medium, which consisted of α minimal essential medium (α-MEM; Lonza, Slough, Berkshire, UK) supplemented with 10% fetal calf serum (FCS), 2 mM l-glutamine, and 100 μg/ml penicillin/streptomycin. Cells were maintained at 37°C in a 95% air-5% carbon dioxide (CO_2_) humidified atmosphere, and medium was changed every 2–3 d. For all experiments, MLO-A5s were seeded at a density of 25,000 cells/well on gelatin-coated 6-well plates containing 2 ml of basal α-MEM and supplemented with ascorbic acid-2-phosphate (50 μg/ml) and β-glycerophosphate (5 mM) at d 1 of culture. Medium was changed every 2–3 d. All reagents were obtained from Sigma-Aldrich (Gillingham, Dorset, UK) unless stated otherwise.

### Primary cilia visualization

Primary cilia are composed of axonemes containing acetylated α-tubulin and were visualized in formalin-fixed MLO-A5 cells by immunostaining with a monoclonal anti-acetylated α-tubulin antibody (produced in mice; clone 6-11B-1, 1 μg/ml; Sigma-Aldrich) for 24 h at 4°C. Cells were washed, and then biotinylated anti-mouse IgG (H+L; raised in goat; 2 μg/ml) was applied for 1 h at 20°C, followed by fluorescein isothiocyanate (FITC)-conjugated streptavidin (10 μg/ml; Vector, Peterborough, UK) for 30 min at 20°C. Cells were counterstained with DAPI 4′-6-diamidino-2-phenylindole (DAPI; 1 μg/ml; Sigma-Aldrich) for 15 min at 20°C to visualize cell nuclei. Cells were visualized using either an Image Express epifluorescent microscope (Axon Instruments/Molecular Devices, Union City, CA, USA) or a Zeiss Axioskop 2 FS MOT laser-scanning confocal upright microscope equipped with a LSM 510 Meta detector (Carl Zeiss Microscopy GmbH, Göttingen, Germany) and a Chameleon titanium:sapphire tunable multiphoton laser (Coherent, Santa Clara, CA, USA).

### Hyaluronan (HA) staining of glycocalyx

To verify that the proteoglycan cell coat (glycocalyx) was continuous around the primary cilium, the HA component of the glycocalyx was stained. Cells were fixed in formalin and stained with biotinylated hyaluronic acid binding protein (5 μg/ml; Merck, Nottingham, UK) for 3 h. Cells were washed, and then streptavidin-FITC (2.5 μg/ml; λ_ex_ 490 nm, λ_em_ 523 nm) was added for 30 min before washing again.

### CH exposure

The drug CH (Sigma-Aldrich) was used to disrupt or remove primary cilia from MLO-A5 cells. Cells were exposed to medium containing CH at final concentration of 4 mM (10 μl/ml of stock solution consisting of 0.4 M CH in distilled water) for 24, 48, or 72 h, followed by a recovery period whereby cells were washed once with PBS and cultured in fresh medium containing no CH. Controls (CH 0) were subjected to a medium change only. Cells were then fixed and labeled with anti-acetylated α-tubulin either immediately or after CH exposure or after 24, 48, or 72 h recovery in fresh medium.

### Application of OFF

**Figure 1.**
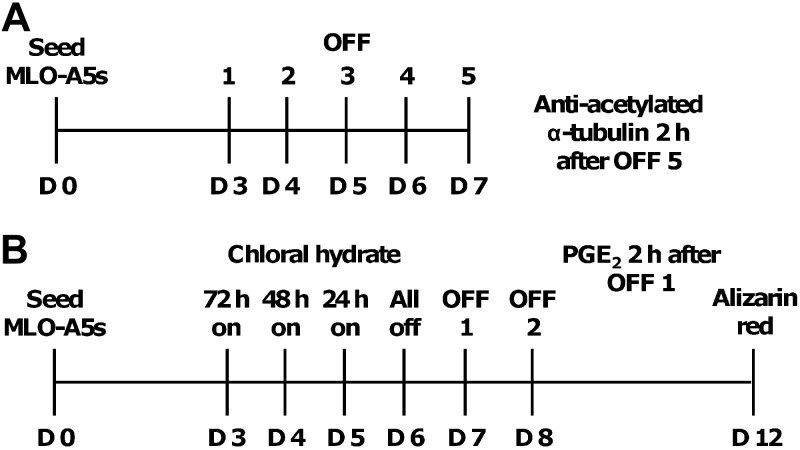
OFF experimental timelines. MLO-A5 cells cultured in gelatin-coated 6-well plates were subjected to 2 separate regimens on a rocking platform to study the effect of OFF on primary cilia morphology (*A*) and the effect of primary cilia removal on OFF-induced mineral deposition (*B*).

Cells were cultured under static (no-flow) conditions or subjected to OFF using a STR6 platform rocker (Stuart Equipment, Stone, UK) with a maximum tilt angle of 6°. Conditions for all OFF experiments were 2 ml of culture medium/well with rocking at the maximum tilt angle at 0.75 Hz for 2 h/d. Two separate regimens were used for studying the response of primary cilia to OFF, as shown in **Fig. 1**. Regimen 1 was used to study primary cilia morphology in response to successive sessions of OFF (d 3–7), and cells were fixed and labeled with anti-acetylated α-tubulin for imaging 2 h after OFF at d 7. Regimen 2 was used to observe whether primary cilia played a role in load-induced mineral deposition of MLO-A5s. Cells were exposed to 4 mM CH for 0, 24, 48, or 72 h, followed by a recovery period of 24 h in fresh medium, and then cells of each CH group were subjected to 2 sessions of OFF on consecutive days (d 7–8) or statically cultured. Medium samples were collected 2 h after the first session of OFF on d 7 for PGE_2_ analysis, and then cells were assayed at d 12 for calcium deposition.

### Total DNA

To monitor any effects of OFF on cell number, Total DNA was measured at d 7 of culture using a fluorescent Quant-iT PicoGreen dsDNA reagent assay kit (Invitrogen Life Technologies, Paisley, UK). Briefly, cells were washed with PBS and then covered with a known volume of a carbonate lysis buffer solution before being scraped and pipetted into microtubes. Samples were stored at 4°C for 24 h. Samples were freeze-thawed 3 times before a known volume of cell lysate was added to the provided Tris-buffered EDTA solution. The Quant-iT PicoGreen reagent, which binds to the double-stranded DNA in solution, was then added, and fluorescence intensity was recorded using a FLx800 microplate fluorescence reader (BioTek, Potton, UK) using 485-nm excitation and 520-nm emission. Total DNA was converted to ng DNA/sample from a standard curve.

### PGE_2_ release analysis

Cells subjected to OFF regimen 2 were incubated in fresh medium for 2 h after the first bout of OFF before collection of the medium. PGE_2_ release into the medium by MLO-A5 cells was determined using a PGE_2_ assay kit (Parameter; R&D Systems, Abingdon, UK) following the manufacturer's protocol. Briefly, the assay is based on the forward sequential competitive binding technique in which PGE_2_ present in the sample competes for horseradish peroxidase (HRP)-labeled PGE_2_ for a limited number of binding sites on a mouse monoclonal antibody. PGE_2_ in the sample was allowed to bind to the antibody in the first incubation. During the second incubation, HRP-labeled PGE_2_ bound to the remaining antibody sites. Following a wash to remove unbound material, a substrate solution was added to the wells to determine bound enzyme activity. The color development was stopped, and the absorbance was read at 405 nm (*A*_405_). Color intensity was inversely proportional to the concentration of PGE_2_ in the sample.

### Extracellular calcium deposition

Alizarin red S (AR) dye (Sigma-Aldrich) binds to Ca^2+^ ions to form a strong red complex and is an indicator of calcium deposition in mineralizing cells and tissues. Samples were fixed with 3.7% formaldehyde followed by diH_2_O washes to remove any non-cell-produced calcium ions. AR solution (5mg/ml, pH 4.1) was applied to fully cover each sample and placed under mild shaking for 15 min at room temperature. Excess dye was washed clear using diH_2_O, and samples were allowed to air dry in a clean environment before qualitative analysis. For quantitative analysis, samples were destained with a known volume of 5% perchloric acid under mild shaking for 15 min, resulting in a clear, dark yellow solution. Absorption was measured at 405 nm using a 96 well microplate reader (BioTek).

### Statistical analysis

All experiments were performed with triplicate samples and carried out 2 or 3 times. Primary cilia counts were performed manually using Image Processing and Analysis in Java (ImageJ) software (U.S. National Institutes of Health, Bethesda, MD, USA). Statistical analysis was performed using GraphPad Prism 5 software (GraphPad, San Diego, CA, USA) and comparisons between sample means for static- and OFF-cultured cells were investigated using a Student's unpaired *t* test. All graphs are plotted as means ± sd, and statistically significant differences are marked. Values of *P* < 0.05 were considered significant.

## RESULTS

### Primary cilia identification in MLO-A5 cells

**Figure 2.**
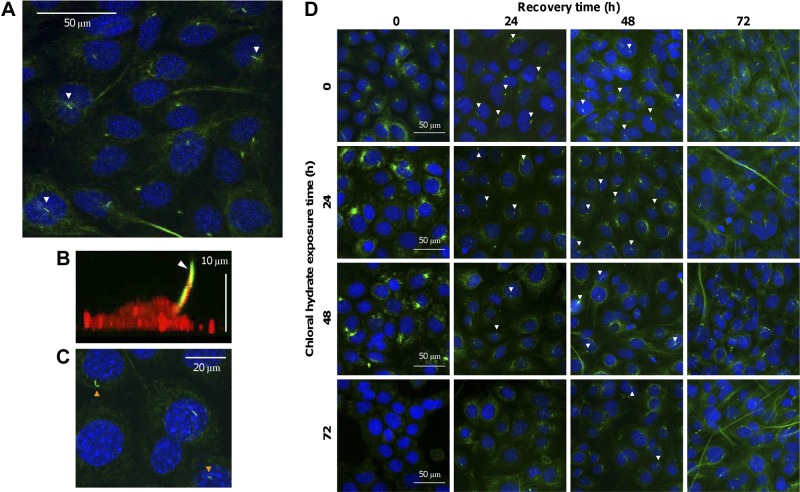
MLO-A5 bone cells express primary cilia. MLO-A5s were stained with anti-acetylated α-tubulin to visualize primary cilia (indicated by white arrowheads) and DAPI to indicate nuclei (blue; *A*, *C*, *D*) or biotinylated HA-binding protein (red; *B*). The primary cilium was seen to protrude from the apical cell surface of MLO-A5 cells cultured *in vitro* (*A*, *B*). The microtubule network within the cell was also visualized (green; *A*). The HA component of the glycocalyx was evident over the entire cell membrane, including around the primary cilium (*B*). Exposure to CH for 24 h caused structural defects in primary cilia of MLO-A5 cells, indicated by orange arrows (*C*), and disorganization of the cell microtubule network, but this recovered after 24 h. As the exposure time of MLO-A5 cells to CH increased from 0 to 72 h, a greater number of primary cilia were damaged or removed (*D*), and the cilia of cells subjected to longer CH exposure also took longer to grow back to normal size when recovering in fresh medium (white arrowheads are representative of relative number of primary cilia present).

To identify primary cilia in MLO-A5 cells and to confirm that they extend from the cell into the extracellular environment, MLO-A5s were labeled with anti-acetylated α-tubulin (**Fig. 2**) due to cilia microtubules being abundantly acetylated. Acetylated α-tubulin-positive primary cilia were visualized as small bright protrusions emanating from most cells (67.2±4%), located close to the cell nucleus (Fig. 2*A*). The microtubule network within the cell cytoplasm was also visualized, but with less intensity, and was seen to be well organized and surrounding the cell nucleus. Confocal images were collected at different points in the *z* plane, and 3-dimensional projections showed that primary cilia extended beyond the apical cell surface (Fig. 2*B*) and were up to 8 μm in length. Staining of the HA component of the cell glycocalyx showed that the primary cilia membrane was continuous with the cell membrane (Fig. 2*B*).

### Damage and removal of primary cilia *via* CH exposure

CH was used to damage and remove primary cilia from MLO-A5 cells by disrupting microtubules. Short-term CH exposure (24 h) caused the microtubule network within the cell to become disorganized; however, this appeared to have reorganized and adopted its original appearance after 24 h in fresh medium (Fig. 2*C*). This was also the case for MLO-A5s exposed to CH for 48–72 h. Exposure to CH for 24 h resulted in a 33% reduction in primary cilia and caused some primary cilia to possess a structural impairment, such as a kink (Fig. 2*C*). As CH exposure time was increased, more primary cilia were removed, and at 72 h exposure nearly 100% had been removed (Fig. 2*D*). The longer the CH exposure time, the longer it took for the number of primary cilia to return to normal levels (CH 0 h; **Table 1**), and after 72 h recovery time in fresh medium, most cells possessed a primary cilium regardless of duration of CH exposure.

**Table 1.** Percentage of cells with a primary cilium after varying CH exposure and subsequent recovery in fresh medium for 24 and 48 h

Recovery time (h)	Chloral hydrate exposure time (h)			
	0	24	48	72
24	67 ± 4	42 ± 5	20 ± 1	3 ± 1
48	66 ± 4	61 ± 3	33 ± 3	17 ± 3

Total cells for each condition: *n* ≥ 450, means ± sd from 3 experimental repeats.

### OFF regulation of MLO-A5 primary cilia

**Figure 3.**
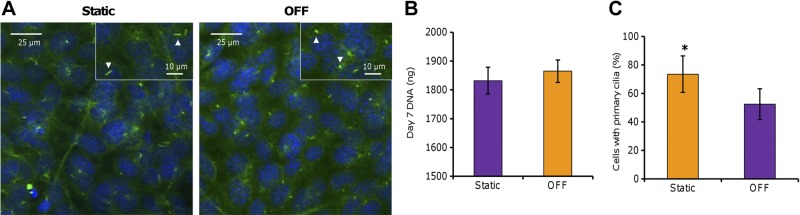
Fluid flow reduces the number of MLO-A5 cells expressing primary cilia. MLO-A5 cells were labeled with anti-acetylated α-tubulin (green) and DAPI (nuclei; blue) after 7 d of culture under static conditions or 5 consecutive days (d 3–7) of OFF (*A*) and assayed for total DNA (*B*) and percentage of cells expressing cilia (*C*). Cells subjected to OFF appear to have shorter primary cilia compared with statically cultured cells, indicated by arrowheads in insets (*A*). Data are means ± sd for *n* = 6 for DNA (*B*) and *n* ≥ 600 cells from 3 experimental repeats for percentage of cells with cilia (*C*). *P < 0.05.

The application of OFF for 5 consecutive days affected the number of primary cilia present in MLO-A5 cells, as well as primary cilia morphology. At d 7, cells cultured under static conditions generally appeared to have longer, more defined primary cilia than those subjected to OFF (**Fig. 3*A***), which overall were less well defined and more stub-like. OFF exposure significantly reduced the number of cells expressing primary cilia by nearly 30% (Fig. 3*C*). There was no difference in total DNA between statically cultured cells and cells subjected to OFF at d 7 (Fig. 3*B*), suggesting that the observed responses are not due to higher cell number or more proliferating cells in the OFF group. This suggests that primary cilia are responsive to OFF.

### Primary cilia regulation of OFF-induced calcium deposition

**Figure 4.**
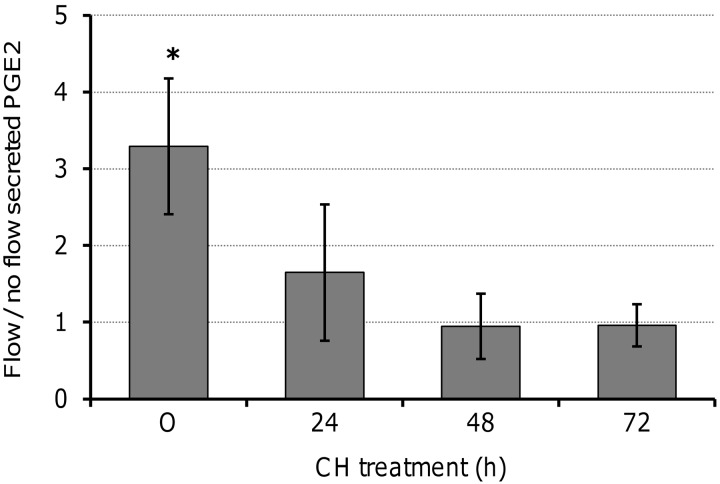
Damage and removal of primary cilia affects the ability of MLO-A5 cells to secrete PGE_2_ in response to OFF. Cells were exposed to CH (0–72 h) and then allowed to recover in fresh medium for 24 h before continued static culture or 2 h of OFF treatment; cells were then cultured for 2 h in fresh medium before collecting media for PGE_2_ analysis. Data are means ± sd (*n*=4–5). *P < 0.05 *vs.* respective static sample.

The cytokine PGE_2_, a regulator of bone metabolism, is released in response to loading by bone cells *in vivo* and *in vitro*, while extracellular mineral deposition, the major inorganic component of bone, is also load-dependent. To determine whether both of these OFF-induced processes required primary cilia, MLO-A5s were exposed to CH for 0–72 h, followed by 24 h in fresh medium, before exposure to 2 sessions of OFF or static culture. After one period of OFF, cells not exposed to CH produced 3-fold more extracellular PGE_2_ compared with statically cultured counterparts (**Fig. 4**). Following the application of CH, no statistically significant differences were found in the level of extracellular PGE_2_ between cells subjected to OFF or their static counterparts. The application of OFF to cells exposed to CH for 24 h resulted in 1.6-fold higher PGE_2_ levels, while for cells exposed to CH for 48–72 h, there was no effect of OFF.

**Figure 5.**
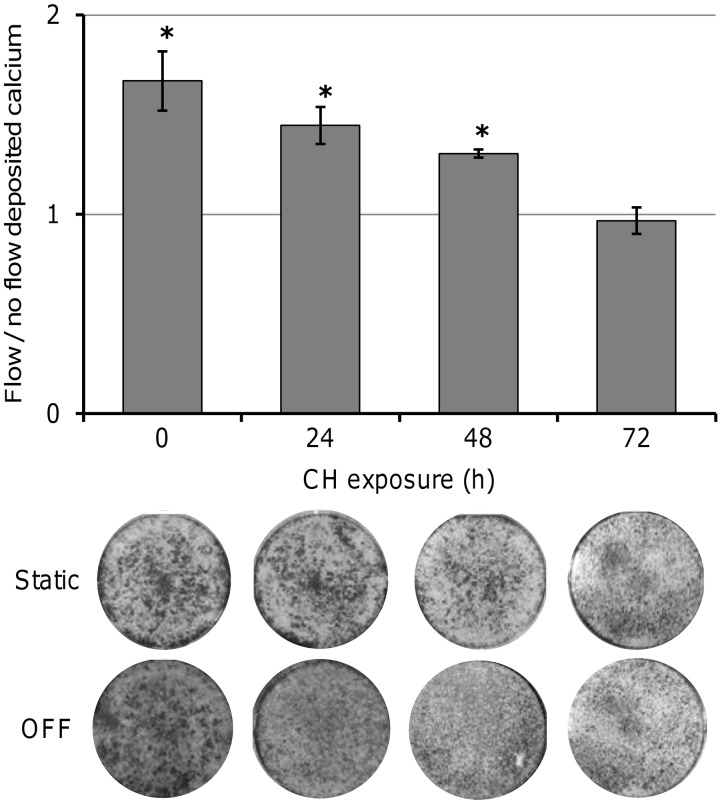
Damage and removal of primary cilia affects the ability of MLO-A5 cells to deposit calcium in response to OFF. Cells were exposed to CH (0–72 h), and then allowed to recover in fresh medium for 24 h before continued static culture or 2-h bouts of OFF as indicated in Fig. 1*B*. Calcium deposition was visualized by AR staining (field of view is entire 6-well plate) at d 12 and quantified by destaining and colorimetry. Data are means ± sd (*n*=9). *P < 0.05 *vs*. respective static samples.

At d 12 of culture, deposited calcium was significantly higher for MLO-A5 cells with no CH exposure that had been subjected to 2 sessions of OFF compared with static counterparts (**Fig. 5**). However, cells exposed to CH for 72 h showed no significant increase in the amount of calcium deposited in response to OFF. Deposited calcium for OFF-exposed cells treated with CH for 0, 24, 48, and 72 h was 1.66, 1.44, 1.31, and 0.97 (respectively) times the level of their static counterparts. The amount of calcium produced in response to OFF was reduced by 33% with CH 24 h, 50% with CH 48 h, and was completely removed with CH 72 h compared to cells exposed to no CH. Although for cells exposed to 24 and 48 h CH, the effect of OFF on calcium deposition was statistically significant, for cells exposed to CH for 72 h, there was no significant difference between OFF treatment and static control treatment. AR staining showed that cells subjected to OFF had a darker and more uniform stain than static control samples, which were patchier, with the exception of cells exposed to CH for 72 h and OFF, which also showed patchy staining. These results indicate that primary cilia are required for OFF-induced calcium deposition.

## DISCUSSION

Mechanical sensitivity is essential for maintaining bone homeostasis and physiological function. However, little is known about the exact mechanisms by which mechanical forces regulate ECM production and resorption in bone cells. Numerous previous studies have identified integrin signaling, the cytoskeleton, the glycocalyx, and transmembrane ion channels all to be involved in mechanosensation (4, 26–28). Recent evidence from both *in vitro* (5, 10) and *in vivo* (29–31) studies have suggested that the primary cilium is also a key mechanosensor in bone. However, to our knowledge, a direct link between the ability of the primary cilium to detect fluid flow and *in vitro* matrix formation by bone cells in response to flow has not previously been established. Here we showed that an intact primary cilium is required for flow-induced deposition of calcium matrix, indicating that the primary cilium would indeed have a role in regulating mechanically induced bone formation in an *in vitro* culture procedure such as bone tissue engineering. After 2 sessions of OFF, calcium deposition was increased compared with static controls, and this flow-induced response was reduced with increasing CH exposure time until it was abolished with CH 72 h.

In this study, the application of OFF for 5 consecutive days appeared to cause a reduction in the length of primary cilia in MLO-A5 cells compared with statically cultured primary cilia, which were longer and more defined. In addition, fewer cilia per cell could be visualized after these repeated bouts of OFF. It has been proposed that the cilium adjusts its sensitivity to the load it receives, either to avoid overloading or in order to detect minor forces. Chondrocytes seeded in a 3D agarose culture model were subjected to cyclic compressive strain, and chondrocyte primary cilia were found to reduce in incidence and length compared with free-swelling culture (19). In another study, freshly harvested rat tail tendons were stress deprived and after 24 h demonstrated an increase in cilia length compared with fresh controls (20). Stress-deprived tendons were then subjected to 24 h of cyclic loading, and cilia length returned to normal levels. Liu *et al.* (32) developed a model for fluid flow around an array of primary cilia and calculated that the drag force and torque experienced were significantly greater in a cilium of 8 μm length compared with one of 2.5 μm. These results support the hypothesis that cilia can adjust their length in order to regulate their mechanosensitivity (33).

Correct function of the primary cilium relies on its structural integrity, defects in this sensory mechanism have been associated with a number of diseases, including many cystic renal disease (34) and cancer (35), but it may also play an important role in the development of musculoskeletal diseases, such as osteoarthritis (36) and osteoporosis (30). In this study, when the CH exposure time was increased, more primary cilia were damaged and completely removed. The partial inhibition of response with shorter durations of CH treatment, which did not completely remove primary cilia, indicates that structural integrity of the primary cilia is essential for proper function.

Another possible mechanism by which primary cilia are able to tune their sensitivity to the extracellular environment is by altering their flexural rigidity in response to load. One of the major differences between microtubules within the cytoplasm and the primary cilium is that ciliary microtubules undergo reversible post-translational modifications, including acetylation and phosphorylation (37). Microtubule-associated protein 1 (MAP1) has been shown to localize to the axoneme of the cilium. The occurrence of MAPs is known to coincide with acetylation and the binding of MAPs has been shown to increase flexural rigidity of microtubules as much as 8-fold (38). Recently, Geiger *et al.* (39) demonstrated that physiological levels of cyclic stretch *in vivo* and *in vitro* result in a significant increase in acetylation of microtubules in a magnitude and duration dependent manner, due to a decrease in histone deacetylase 6 (HDAC6) activity.

For bone tissue engineering, understanding which early time point signals correspond to downstream matrix formation is a key step for monitoring the production of a suitable bone construct for transplantation. However, the relationships between early and late responses to mechanical load remain unclear. PGE_2_ is released by osteoblasts and osteocytes after mechanical stimulation *in vivo* and *in vitro* with the primary role of regulating bone metabolism (4). It is not always easy to predict the long-term outcome of secretion of PGE_2_, as it stimulates both bone formation and resorption *in vivo* and osteoclasts and osteoblasts *in vitro* (40). However, PGE_2_ does seem to consistently stimulate osteoblast differentiation *in vitro*. Malone *et al.* (10) observed that the load-induced increase in extracellular PGE_2_ was abolished when primary cilia formation was inhibited in both immature osteoblasts (MC3T3-E1) and osteocytes (MLO-Y4). In this study, PGE_2_ levels in the medium increased when MLO-A5 cells were subjected to OFF, and this response was removed with the application of CH. While we did not test whether the higher calcified matrix deposition caused by fluid flow was directly mediated by PGE_2_, we do show a strong similarity between the inhibition of the PGE_2_ response (which can be detected a few hours after flow) and the calcified matrix response which takes several days to be detectable.

The relationship between the primary cilium and other demonstrated mechanosensors, such as integrins, and the cytoskeleton remains unclear. However, integrin binding sites have been demonstrated on cilia (41) and the cilium has also been shown to be necessary for actin cytoskeleton reorganization in response to unidirectional flow in endothelial cells (42). Here we show, for the first time, that HA can be detected on the region of the membrane surrounding the primary cilia; this suggests that the cilia is coated by the same glycocalyx that coats the rest of the cell membrane. However, it is important to note that the GAG/PG layer found on cells *in vitro* does not necessarily represent the true *in vivo* thickness of the glycocalyx (43). The glycocalyx is also a proposed mechanotransducer and has been shown to play a sensory role in bone cells, as well as other cell types (9, 44). Using laminar flow, Morris *et al.* (9) induced FSS on mature bone cells, causing an increase in collagen production, a response that was absent on removal of HA. In an earlier study, Reilly *et al.* (44) inhibited the ability of osteocyte-like cells to up-regulate PGE_2_ release in response to flow by removal of HA. This leads to interesting questions about what effect removal of the cilia's HA coat may have on cilia-specific mechanosensory capabilities.

There may well be several other pathways by which fluid-flow induced shear stress is transduced and modulated. When a cell is mechanically stimulated, pronounced cytoskeletal alterations can occur, including actin reorientation, microtubule polymerization/depolymerization, and reorganization of focal adhesion sites. It is thought that cytoskeletal reorganization is partly undertaken to minimize cell internal stresses caused by external stress (28) and this reorganization has been shown to be dependent on the type, magnitude, and duration of the external stress. It has been shown that steady flow applied to osteoblasts results in reorganization of the actin fiber network; however, this did not appear to occur with the application of OFF (28). This is consistent with the possibility that oscillatory fluid shear stress may stimulate different mechanotransduction pathways from steady/dynamic fluid shear stress. It is likely that multiple mechanosensation mechanisms are present in bone cells and that these mechanisms are activated at different levels of stimulus. Multiple response mechanisms would be advantageous in terms of biological redundancy or may be associated with distinct mechanically regulated functions, such as turnover or repair.

Understanding which signals correspond to downstream matrix formation is a key step for controlling the production of a suitable construct for transplantation. Cultured kidney cells exposed to fluid flow showed a primary cilium dependent extracellular Ca^2+^-dependent intracellular Ca^2+^ release (18). This cilium-mediated Ca^2+^ entry also required the stretch-activated ion channel polycystin 2 (45). In bone cells, flow-induced Ca^2+^ flux was shown to be independent of primary cilia (10), and inhibition of polcystin 2 also did not remove the flow-induced flux of Ca^2+^; however, cyclic adenosine monophosphate (cAMP) production in response to flow was cilia dependant (46). Interestingly, polycystin 1-knockout mice exhibited a reduced ability to respond with increased bone formation to an external mechanical force, and osteoblasts from these mice did have a reduced ability to respond to fluid flow *in vitro* with an intracellular Ca^2+^ signal (47). In chondrocytes, the primary cilium is required for compression-induced Ca^2+^ signaling mediated by ATP release, but while cilia were not shown to be the initial mechanoreceptors, they were required for downstream ATP reception (19). These findings suggest that different cell types contain distinctly different mechanisms that are responsible for cilium-mediated mechanosensation.

Most of the work to date concerning the primary cilium and its role as a mechanosensor has focused on mature/differentiated cells. However, human MSCs (hMSCs) and osteogenic progenitor cells, commonly advocated as potential autologous cell sources for bone tissue engineering, also possess a primary cilium (48, 49). A recent study by Hoey *et al.* (50) suggests that increases in short-term osteogenic responses of human bone marrow MSCs (hbMSCs) to OFF are also dependent on a fully functioning primary cilium. We used an osteoblastic cell line MLO-A5 for these studies due to the ability of these cells to rapidly form bone matrix *in vitro* (23); however, in all the *in vitro* systems we have tested either primary hMSCs or human embryonic mesenchymal progenitors have responded to mechanical load in the same way as MLO-A5s, albeit with less matrix formation at the same time-points (6, 22, 51). It has also been shown that bone cell sensitivity to mechanical forces is dependent on the stage of bone cell maturity (*i.e.*, immature-mature osteoblast or immature-mature osteocyte; refs. 52, 53), and often osteogenic progenitor cells do not respond to mechanical forces without the presence of differentiation medium (54). Addressing the role that primary cilia play at different stages of cell differentiation, including undifferentiated progenitor cells, immature osteoblastic cells, and fully differentiated osteocytes would be an interesting target of future studies.

The ultimate aim of bone tissue engineering is to control osteogenic cell differentiation and to produce sufficient amounts of organized load-bearing mineralized matrix in a short amount of time. These experiments were performed in monolayer whereas *in vitro* bone formation/tissue engineering strategies usually involve seeding cells in porous scaffolds. However, in a scaffold with relatively large pores, cells attach to the strut walls in a similar way to their attachment to a 2D surface (9, 55). These similarities mean that the regions of the cell shielded and exposed to flow will probably also be similar, and therefore responses to flow within this rocking-well environment could provide information on the optimization of flow conditions for 3D porous scaffolds in flow bioreactors. In addition to this, while the primary aim of this study was to examine the role of the primary cilia in *in vitro* bone-like tissue formation, studies such as this provide potential targets for *in vivo* investigation of mechanically induced bone formation. The relationship between the cell's ECM and surrounding fluid differs in a monolayer environment compared with that of the 3D *in vivo* environment; given that the space between the cell membrane and the bone matrix for an osteocyte *in vivo* is very small and filled with proteoglycans, an osteocyte's primary cilia may not be able to deflect in response to flow in the way that it can in a flow chamber or rocking culture dish. It has been observed *ex vivo* that primary cilia project into the ECM of a variety of musculoskeletal tissues (56), where they have also been shown to interact with the ECM proteins in cartilage (57), as well as adopting an orientation parallel to the collagen fibrils in tendon (58). Similar circumstances may occur in bone, whereby integrins on the primary cilia may sense deformations of the ECM and convert these into biological signals.

Although it is not yet known whether it is a flow-mediated effect, there is certainly mounting evidence that the primary cilia are involved in the mechanoresponsiveness of bone *in vivo* and may therefore be a mediator of skeletal homeostasis (59). Temiyasathit *et al.* (30), showed that the response of bone formation to load was reduced in Kif3a-knockout mice, while Qui *et al.* (31) observed reduced bone mineral density and development of osteopenia in Kif3a-null mice. Kif3a is an essential subunit of the kinesin II IFT motor protein, and its absence causes disruption of IFT and so disruption of formation, maintenance, and function of primary cilia (30). Deletion of Kif3a in mouse preosteoblasts around an intraosseous implant resulted in a failure of the mice to produce and organize collagen in response to load (29).

## CONCLUSIONS

Understanding the mechanisms behind how a cell senses a mechanical force and converts this into a biological response is important for tissue engineering strategies involving the use of mechanical stimulation. Identifying the pathways involved in mechanotransduction responses of bone will also aid research into fracture healing, osseointegration, and clinical treatments of bone disorders where it is still unclear what the optimal mechanical loading and exercise regimens are. The present study showed that bone cell primary cilia adjust their morphology in response to OFF in an apparent attempt to alter sensitivity to loading. Mineralized matrix deposition is the end stage of bone cell differentiation, and the primary cilium seems to play an essential role in load-induced bone matrix formation. The evidence presented here for primary cilia as a mechanosensor in bone cells highlights that they could be targeted for optimizing loading regimes and controlling the subsequent production of *in vitro* bone matrix and may inform clinical treatments of bone disorders caused by dysfunctional responses to loading.
